# Gestational Early-Time Restricted Feeding Results in Sex-Specific Glucose Intolerance in Adult Male Mice

**DOI:** 10.1155/2023/6666613

**Published:** 2023-09-29

**Authors:** Molly C. Mulcahy, Noura El Habbal, Detrick Snyder, JeAnna R. Redd, Haijing Sun, Brigid E. Gregg, Dave Bridges

**Affiliations:** ^1^University of Michigan School of Public Health, Department of Nutritional Sciences, Ann Arbor, MI, USA; ^2^Michigan Medicine, Department of Pediatrics, Division of Diabetes, Endocrinology and Metabolism, Ann Arbor, MI, USA

## Abstract

The timing of food intake is a novel dietary component that impacts health. Time-restricted feeding (TRF), a form of intermittent fasting, manipulates food timing. The timing of eating may be an important factor to consider during critical periods, such as pregnancy. Nutrition during pregnancy, too, can have a lasting impact on offspring health. The timing of food intake has not been thoroughly investigated in models of pregnancy, despite evidence that interest in the practice exists. Therefore, using a mouse model, we tested body composition and glycemic health of gestational early TRF (eTRF) in male and female offspring from weaning to adulthood on a chow diet and after a high-fat, high-sucrose (HFHS) diet challenge. Body composition was similar between groups in both sexes from weaning to adulthood, with minor increases in food intake in eTRF females and slightly improved glucose tolerance in males while on a chow diet. However, after 10 weeks of HFHS, male eTRF offspring developed glucose intolerance. Further studies should assess the susceptibility of males, and apparent resilience of females, to gestational eTRF and assess mechanisms underlying these changes in adult males.

## 1. Introduction

Behaviors that impact circadian rhythms, such as sleep, light exposure, and shift work, have long been associated with human health. The circadian rhythm follows a 24-hour cycle which is governed at the cellular level by a transcription factor system [1–3]. This highly coordinated system can be entrained according to external cues. This system imparts a rhythm to many physiological systems, including metabolism [4]. Recently, food intake has been found to impact the oscillations of the circadian rhythm [5].

Recent evidence demonstrates the timing of food intake in reference to circadian rhythms can impact propensity for health or disease [6]. Time-restricted feeding/eating (TRF/E), a method of intermittent fasting, is thought to align caloric intake with naturally occurring circadian rhythms of metabolism [7]. Timing of food intake is capable of influencing metabolic systems for either poor health from chronodisruption or good health with either diurnal or nocturnal feeding, depending on the species [8].

To our knowledge, no estimate of the prevalence of TRE in humans exists. However, according to one sample, up to ten percent of people surveyed that stated they followed a diet in the year 2020 had attempted “intermittent fasting,” making it the most prevalent dietary intervention in that sample [9]. There are critical periods of development in the lifespan where changes to dietary behaviors can impact current and future health status. One such critical period is pregnancy. During pregnancy, habitual timing of food intake may be altered for many reasons: religious practice, food insecurity, disordered eating behaviors, nausea and vomiting of pregnancy/morning sickness, changes in taste/food preferences, or intentional timing of eating for weight maintenance. Very little research has evaluated the timing of eating during pregnancy and its impact on offspring health. One cross-sectional analysis found that extending the overnight fast during pregnancy was associated with lower blood glucose levels at midgestation [10]. Another recent work demonstrated that up to 23.7% of a human pregnant and recent postpartum cohort said they would be willing to try TRE during pregnancy [11]. However, there is currently no information on the long-term implications of this dietary strategy for progeny. The most available literature examines fasting during the month of Ramadan while pregnant. A review of these studies found that children born to those who fasted during pregnancy have similar birth weights and rates of preterm birth as those who did not fast [12]. In a recent review, Ramadan exposure *in utero* was associated with a smaller body size and stature in later periods of life [13]. However, these studies are limited, and Ramadan fasting is an imperfect model for TRF, as food intake is not only limited in duration but also not permitted during the normal active phase for humans.

There is much interest in the TRE diet, and interruptions in food intake are known to occur during pregnancy; however, research about the effects of intentional fasting during pregnancy is limited to the observance of Ramadan, a cross-sectional study about attitudes toward the practice [11], and one case report of fasting to improve gestational diabetes [14]. Detailed modeling of TRF in pregnancy is warranted, as TRE is currently thought to exist in human populations [11, 14], yet long-term effects are unknown.

Other groups have demonstrated that the circadian rhythm and entrainment with external cues, such as phase shifts in lighting, during gestation can affect perinatal health outcomes in rodent models. In fact, chronic use of photoperiod shifts during gestation and early postnatal life in rats can result in altered oscillations of hormones and behaviors in dams, to impact gestational age and birth weight, and to cause endocrine abnormalities, elevations in mean glucose, and glucose intolerance in adult male offspring [15]. Others have found worsened glycemic health in both male and female adult offspring with chronodisruption despite no differences in body weight or litter size [16]. This is important because it demonstrates that external cues impact health outcomes during pregnancy. Light cues are the most powerful zeitgebers, but other external cues such as the timing of food intake have not been investigated in pregnant populations.

Previous studies of the maternal diet during pregnancy have focused on dietary restriction or macronutrient excess in pregnancy, with little-to-no attention directed toward temporality of food intake. At the time of this manuscript, two studies of TRF during pregnancy in rodents exist. The first emphasized fetal health and was completed in the context of preventing complications from a high-fat, high-sucrose diet (HFHS) during gestation in a rat model. Upadhyay et al. found that 9-hour TRF improved fetal lung development [17] and placental oxidative stress markers [18] at embryonic day (E) 18.5 compared to *ad libitum* fed dams. This approach did not evaluate the long-term, postnatal effects of TRF, and the independent effects of TRF are complicated by the use of a high-fat, high-sucrose diet. The second, also in rats, evaluated 12-hour access in light and dark cycles to a chow diet during pregnancy and followed male and female resultant offspring until 150 days of age [19]. Adult female offspring of dams fed in the dark cycle with TRF were found to be glucose intolerant *in vivo* and have reduced glucose-stimulated insulin secretion *in vitro* in both male and female offspring islets and altered glucose metabolism in adult offspring of TRF-fed dams [19]. However, this study only assessed offspring body composition at birth and once during adulthood. It also did not evaluate glycemic health until late adulthood, leaving the developmental trajectory of gestational eTRF exposed offspring unexamined.

The effects of TRF in nonpregnant human populations are inconsistent. Similarly, insulin sensitization results in some [20–24], but not all trials of TRF [25, 26]. The duration and timing of feeding windows for TRF employed in human can vary. Lengths of feeding windows can vary between 4 [20] and 12 hours [27], and the feeding window can be early [21–23, 28] vs. late [21, 26, 29] in the day, control of caloric intake isocaloric [23] vs. unrestrained caloric intake [25, 26, 30], and inpatient observation [23] or outpatient adherence monitoring [25, 26]. As such, the biological effects of this eating strategy are not clear, even in nonpregnant humans.

Results from rodent models of TRF are more consistent than those of human trials. These have found TRF of an HFHS diet reduces body weight compared to ad libitum feeding [31–36], can improve the homeostatic model assessment for insulin resistance (HOMA-IR) [33, 36, 37], and may limit complications such as insulin resistance [34, 35] from HFHS feeding.

Taking together the likelihood that food intake can be time-disrupted in pregnancy and the evidence of TRF being a potent method to improve body composition and glycemic health in adult mice, we sought to evaluate the impact of TRF of normal laboratory chow (6-hour, early dark cycle) before and during pregnancy on resulting offspring body composition and glycemic health through adulthood.

## 2. Methods

### 2.1. Animal Care and Use

Male and female C57BL/6J mice were obtained from Jackson Laboratory (RRID IMSR_JAX:000664). All animals were maintained on a 12-hour light/dark (12 dark (ZT12, 6pm): 12 light (ZT0, 6am); ZT = zeitgeber time) cycle in a temperature (70°C) and humidity (40–60%)-controlled room. After one week of acclimatization, females were single-housed for the remainder of the experiment and males were socially housed until mating. Dams and sires were randomized to either early time-restricted feeding (eTRF) or *ad libitum* (AL) feeding (dams n 8 = eTRF, 9 = AL). This study was completed in two independent cohorts of animals. The phenotypes noted in offspring were highly consistent between cohorts, and we found no statistical effect modification by cohort (data not shown). Therefore, data shown are the combined total from cohorts one and two, and statistical tests do not include effects of the cohort in the model. Dams and sires that were fed AL had 24-hour access to a chow diet (NCD, PicoLab Laboratory Rodent Diet, 5L0D; 5% of calories from fat, 24% from protein, and 71% from carbohydrates). Dams and sires that were fed eTRF had 6 hours of NCD food access during the early dark cycle (ZT 14-ZT 20). Water was provided *ad libitum* throughout the study to both experimental groups. After one week of either AL or eTRF feeding (beginning age 120 days), age-matched sires were introduced into cages for breeding. Males were kept in the cage until a copulatory plug was detected. Daily, dams were transferred to a clean cage at ZT20, allowing for a cage free of food for eTRF animals and similar levels of handling between experimental groups. After birth, all dams switched to AL and were maintained on this diet until weaning at postnatal day (PND) 21.5. Therefore, any phenotype in the offspring is attributable to modifications to the pregestational/gestational diet. All experimental protocols were reviewed and approved by the University of Michigan Institutional Animal Care and Use Committee.

### 2.2. Offspring Growth and Food Intake Monitoring

Pups born were weighed and counted within 24 hours of birth. Litters were reduced to 4 pups (2 male and 2 female, when possible) at PND 3.5 to standardize milk supply between litters. At PND 21.5, offspring were weighed and body composition was assessed using EchoMRI 2100 (EchoMRI) before being weaned by sex and maternal-feeding regimen and housed 4-5 per cage (eTRF males = 11, eTRF females = 19, AL males = 16, and AL females = 17). Offspring were given AL access to NCD until PND 70. Food intake and body composition were assessed weekly. Food intake is represented as an average per animal per day. To correct to food spillage, during weekly food measurements, cages were examined for excessive levels of pellet shredding or food loss from the hopper. Cages meeting these criteria for spillage were excluded from statistical analysis for that week. After PND 70, all animals began an AL 45% high-fat, high-sucrose diet (HFHS; Research Diets D12451; 45% fat/20% protein/35% carbohydrate). Weekly, body composition and food intake measurement continued during HFHS feeding. Feeding efficiency was calculated for the two periods of diet (NCD and HFD). Fat and lean mass measurements collected via EchoMRI at the beginning of the period were subtracted from the final fat and lean mass measurements for that feeding period. This represented the total gain in fat and lean mass during this diet period. These values were then multiplied by 9 and 4, respectively (Atwater factors for fat and carbohydrate/proteins). The product was then divided by total kcals consumed during the feeding period. The result is expressed as a percentage, where larger numbers represent greater efficiency in turning consumed kcals into bodily tissues (eTRF males = 9, eTRF females = 16, AL males = 14, and AL females = 14).

### 2.3. Insulin Tolerance and Glucose Tolerance Testing

Baseline intraperitoneal insulin (ITT: eTRF males = 9, eTRF females = 17, AL males = 18, and AL females = 19) and glucose tolerance tests (GTT: eTRF males = 4, eTRF females = 4, AL males = 7, and AL females = 6) were assessed at young adulthood toward the end of the NCD diet period (PND 60–70, in that order). Animals were transferred into a cage with no food during the early light cycle (ZT 2), with water freely available. After 6 hours, fasting blood glucose was assessed using a tail clip and a handheld glucometer (OneTouch Ultra). Shortly thereafter, an intraperitoneal injection of insulin was administered (Humulin, u-100; 0.75 U/kg lean mass). Blood glucose was assessed by using a glucometer every 15 minutes for 2 hours. One week later, glucose tolerance was assessed in a similar way (D-glucose, 1.5 g/kg lean mass). Insulin and glucose tolerance was then reassessed after HFHS feeding (PND 140–160: eTRF males = 9, eTRF females = 18, AL males = 18, and AL females = 18) (insulin dose 2.5 U/kg lean mass and glucose dose 1.0 g/kg lean mass). The area under the curve was calculated for each animal by taking the sum of glucose at each time point and then was averaged by sex and a maternal-feeding regimen. Rates of drop for ITT were calculated by limiting the dataset to the initial period after insulin administration (<60 minutes), taking the log of the glucose values and generating a slope for each animal. After each animal's rate of drop was calculated, values were averaged by sex and treatment.

### 2.4. Glucose-Stimulated Insulin Secretion Testing In Vivo

As an exploratory analysis, one week after GTT and ITT, animals underwent intraperitoneal glucose-stimulated insulin secretion (GSIS) testing (PND 160–170: eTRF males = 4, eTRF females = 4, AL males = 5, and AL females = 8). At ZT2, animals were placed in a clean cage without food and with unrestricted access to water. After a 6-hour fast, animals were lightly anesthetized with isoflurane via drop jar and a baseline blood sample was collected via retro-orbital bleed with a heparinized capillary. Following baseline blood collection, an intraperitoneal injection of D-glucose (1.0 g/kg lean mass) was given. After 15 minutes, animals were lightly anesthetized in the same manner and another blood sample was collected. Blood samples were allowed to clot on wet ice (∼20 minutes) and then were spun down in a cold centrifuge (4°C, Eppendorf microcentrifuge, model 5415R) for 20 minutes at 2000*g*. Serum was pipetted off and stored at −80°C until analysis. Serum insulin was assessed via a commercially available ELISA kit (ALPCO 80-INSMSU-E10). Serum insulin was assessed in 5 *μ*L samples and read via a colorimetric assay.

### 2.5. Statistical Analysis

All measures with *p* values <0.05 were considered statistically significant. Data are presented as the mean ± standard error throughout. All statistical analyses were performed using R version 4.0.2 [38]. To minimize potential bias, the analysis plan was chosen prior to the start of experiments and remain unchanged upon data analysis. Repeated measures, such as body composition, cumulative food intake, and responses to GTT or ITT, were assessed via mixed linear-effects modeling with random effects of mouse ID and dam and fixed effects of maternal dietary treatment, age, and sex using lme4 version 1.1–26 [39]. Body composition and food intake were measured repeatedly in two separate conditions: during NCD feeding and after being switched to HFHS. Analyses were tested for significant interactions between sex and maternal dietary treatment. Models were assessed using two-way ANOVA for sex and maternal dietary treatment, with an interaction between the two. If a significant interaction was observed, data were sex-stratified and pairwise comparison was repeated, reporting the effect size and *p* value for the interaction. Otherwise, sex was used as a covariate in a noninteracting model. Observations were tested for normality by the Shapiro–Wilk test and equivalence of variance by Levene's test. Pairwise measures that were normal and of equal variance utilized Student's *t*-tests. Measures that were not normally distributed used nonparametric Mann–Whitney tests.

## 3. Results

### 3.1. Gestational eTRF Increases Food Intake, but Not Body Weight in Early Life

**Figure 1:**
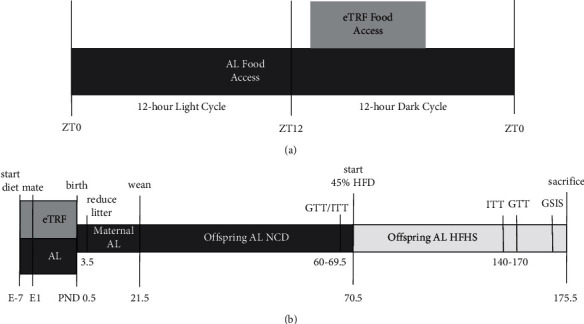
Experimental protocol and timing. (a) Food availability and timing for dams during pregnancy. Food access began at ZT14 for early time-restricted feeding dams (eTRF, light gray, *n* = 8) and continued until ZT20, a total of 6 hours. Food was available 24 hours a day for ad libitum dams (AL, dark gray, *n* = 9). (b) Offspring experimental protocol. After birth, all dams had AL access to laboratory chow (NCD). Litters were reduced to 4 (2 males and 2 females when possible) on postnatal day (PND) 3.5. Offspring were weaned by a maternal-feeding regimen at PND 21 and maintained on AL NCD for 70 days. Weekly, body composition and food intake measurements were taken throughout the experiment. At 70 days of age, insulin tolerance tests (ITT: eTRF males = 9, eTRF females = 17, AL males = 18, and AL females = 19) and glucose tolerance tests (GTT: eTRF males = 4, eTRF females = 4, AL males = 7, and AL females = 6) were conducted before switching all animals to a 45% high-fat, high-sucrose diet (HFHS) with sucrose. Animals were on HFHS for 10 weeks before repeating ITT and GTT (eTRF males = 9, eTRF females = 18, AL males = 18, and AL females = 18) and an in vivo glucose-stimulated insulin secretion test (GSIS: eTRF males = 4, eTRF females = 4, AL males = 5, and AL females = 8). Animals were euthanized after these tests. Abbreviations: zeitgeber time (ZT); ZT0 = lights on; ZT12 = lights off.

To model gestational early time-restricted feeding (eTRF), we used a normal chow diet (NCD) and assigned female mice to either unrestricted (*ad libitum,* AL) or 6 hours of restricted food availability between ZT14-20 (eTRF) (Figure 1(a)). This period represents the active phase of both pregnant and nonpregnant mice [40]. This approach limits potential sleep disruptions and is more translationally relevant to human dietary restriction. This treatment started a week before mating in both dams and sires and continued through delivery (Figure 1(b)). We find no evidence of maternal eTRF causing significantly lower daily food intake during pregnancy, nor are there changes in body weight (Supplementary Figures 1A and 1B). Litters were normalized to equal sizes on postnatal day 3 to reduce variability and effects of lactation.

**Figure 2:**
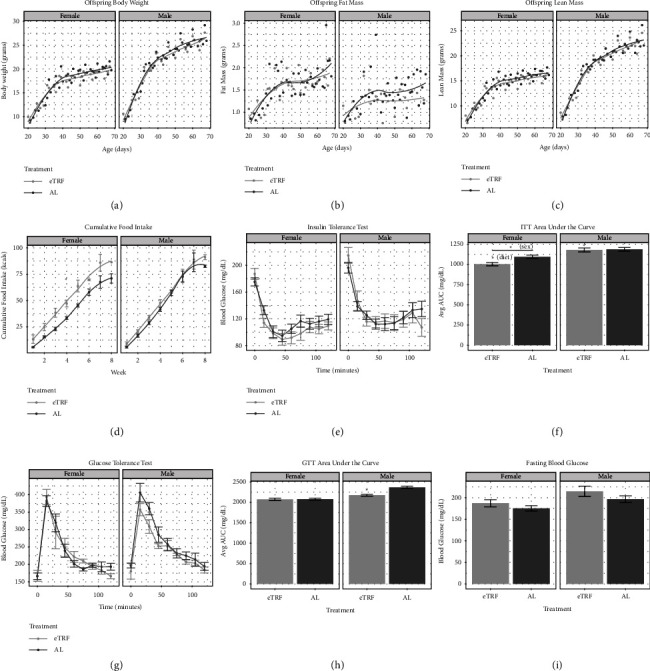
Early life body composition, food intake, and glycemic homeostasis. (a) Body weight in grams from PND21–PND70 in males and females, averaged by age, maternal-feeding regimen, and sex. (b) Fat mass in grams from PND21–PND70 in males and females, averaged by age, maternal-feeding regimen, and sex. (c) Lean mass in grams from PND21–PND70 in males and females, averaged by age, maternal-feeding regimen, and sex. (d) Food intake in kcals per mouse per day, averaged by week, maternal-feeding regimen, and sex. ^*∗*^*p* value <0.05 for diet. (e) Insulin tolerance test (ITT)∼PND 70, averaged by maternal-feeding regimen, sex, and time in minutes. (f) Area under the curve (AUC) for ITT, averaged by maternal-feeding regimen and sex. ^*∗*^*p* value <0.05 for the effect of diet in males. (g) Glucose tolerance test (GTT)∼PNG 70, averaged by maternal-feeding regimen, sex, and time in minutes. (h) AUC for GTT, averaged by maternal-feeding regimen and sex. ^*∗*^*p* value <0.05 for the effect of diet in males. (i) Fasting blood glucose (FBG) PND 70, averaged by maternal-feeding regimen and sex. Animals included in body composition measurements, FBG, ITT, and GTT: *n* = 11 eTRF males, 16 AL males, 19 eTRF females, and 17 AL females. Number of cages in food intake analysis: *n* = 4 eTRF males, 5 AL males, 4 eTRF females, and 5 AL females.

The pups resulting from this experiment were weighed, and their body composition was assessed weekly and then analyzed using linear mixed-effect modeling. We found significant and expected effects of age and sex (older mice weigh more than younger mice, and male pups weigh more than females), but there was no effect modification of maternal eTRF on body weight (Figure 2(a), *p*_diet_ = 0.47), lean mass (Figure 2(c), *p*_diet_ = 0.45), or fat mass (Figure 2(b), *p*_diet_ = 0.47). There was no interaction between sex and a maternal-feeding regimen in cumulative food intake (*p*_diet∗sex_ = 0.38). However, cumulative food intake in the NCD period is 22% higher in eTRF females than in AL females and 10% higher in eTRF males than in AL males (Figure 2(d), *p*_diet_ = 0.016). Assessing the efficiency by which food is converted into stored mass resulted in a 12% reduced feeding efficiency in eTRF female offspring (*p*_sex_ < 0.00001), which is not present in males (Supplementary Figure 2A).

### 3.2. Gestational eTRF Modestly Improves Glucose Tolerance in Young Adult Males

To assess glucose homeostasis in the offspring, we conducted ITTs and GTTs between PND 60 and 70. Male offspring averaged 15mg/dL higher blood glucose during insulin tolerance testing than females (*p*_sex_ = 0.0018), but no effect of maternal dietary restriction was evident through linear mixed-effect modeling (Figure 2(e), *p*_diet_ = 0.73). Summarizing the ITT by calculating the area under the curve (AUC) demonstrated there was no diet : sex interaction (*p*_diet:sex_ = 0.069), but there was an effect of maternal restriction where eTRF offspring had lower AUC than AL offspring, 8.5% and 2.2% lower in females and males, respectively (*p*_diet_ = 0.013), and a significant effect of sex (*p*_sex_ < 0.0001). As expected, males had a higher AUC than females (Figure 2(f)). The initial response to insulin (the rate of glucose decline over the first 60 minutes, not pictured) was not significant for sex (*p*_sex_ = 0.10) or treatment (*p*_diet_ = 0.83). These data suggest that gestational eTRF slightly improves the response to insulin challenge in adult mice and that this is not driven by reduced fat mass.

Glucose tolerance was similar in young adulthood between groups in both males and females (Figure 2(g)). We found no significant effect of diet (*p*_diet_ = 0.53) on the rise in blood glucose during GTT, but there was an effect of sex (*p*_sex_ = 0.0093) on glucose tolerance, again with expected higher glucose levels in male mice. The summarized AUC for the GTT (Figure 2(h)) shows a significant interaction between sex and maternal dietary treatment (*p*_sex:diet_ = 0.00082). eTRF males had an 8.2% lower AUC than their AL counterparts (*p*_diet_ < 0.0001), while this was absent in females (*p*_diet_ = 0.99). Fasting blood glucose, assessed before ITT and GTT, was 10.4% higher in males than in females (*p*_sex_ = 0.0054, Figure 2(i)) but did not differ significantly by maternal dietary treatment (*p*_diet_ = 0.18). Taken together these data suggest that gestational eTRF has very a mild effect on adult offspring, despite the narrow feeding window. Offspring whose mothers were fed eTRF had slightly improved responses to insulin and glucose challenge but no differences in body weight or in fat mass.

### 3.3. HFHS Feeding in Adult Offspring Exposed to eTRF during Gestation Generates Sex-Specific Glucose Intolerance

**Figure 3:**
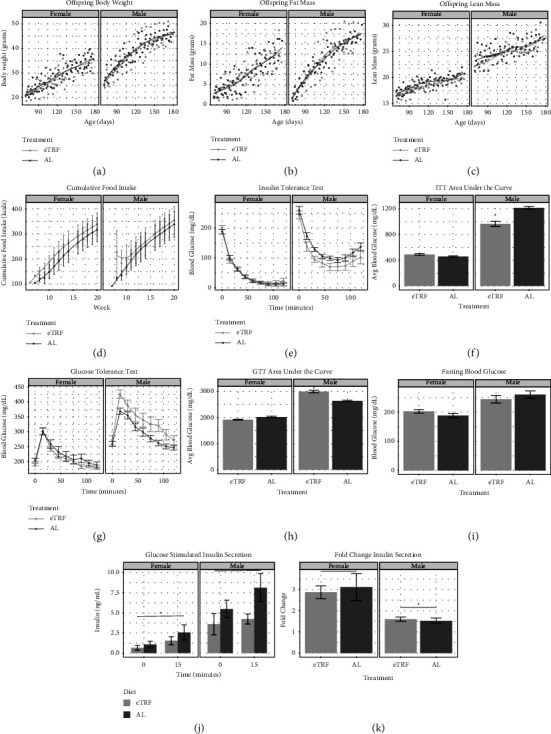
Body composition, food intake, and glycemic response to high-fat, high-sucrose diet feeding in adulthood. (a) Body weight in grams from PND 70 to 175 in males and females, averaged by age, maternal-feeding regimen, and sex. (b) Fat mass in grams from PND 70 to 175 in males and females, averaged by age, maternal-feeding regimen, and sex. (c) Lean mass in grams from PND 70 to 175 in males and females, averaged by age, maternal-feeding regimen, and sex. (d) High-fat, high-sucrose diet (HFHS) intake in kcals per mouse per day averaged by week, maternal-feeding regimen, and sex. (e) Insulin tolerance test (ITT) after 10 week of HFHS, averaged by age, maternal-feeding regimen, sex, and time in minutes. (f) Area under the curve (AUC) for insulin tolerance test, averaged by maternal-feeding regimen and sex. ^*∗*^*p* value <0.05 for diet in males. (g) Glucose tolerance test (GTT) after 10 weeks of HFHS, averaged by maternal-feeding regimen, sex, and time in minutes. (h) Area under the curve (AUC) for GTT after 10 weeks of HFHS, averaged by maternal feeding regimen and sex. ^*∗*^*p* value <0.05 for the effect of diet in males. (i) Fasting blood glucose (FBG) after 10 weeks HFHS, averaged by maternal-feeding regimen and sex. (j) Glucose-stimulated insulin secretion (GSIS), averaged by maternal-feeding regimen, sex, and time. (k) Fold change of insulin secretion during GSIS, averaged by maternal-feeding regimen and sex. ^*∗*^*p* value <0.05 for the effect of sex. Animals included in body composition, FBG, ITT, GTT, and GSIS: *n* = 11 eTRF males, 16 AL males, 19 eTRF females, and 17 AL females. Cages in food intake analysis: *n* = 4 eTRF males, 5 AL males, 4 eTRF females, and 5 AL females.

Given that adult offspring were minimally affected by gestational eTRF exposure, we administered a high-fat, high-sucrose (HFHS) overnutrition challenge, *ad libitum* access to 45% of energy from fat and 17% of energy from sucrose after PND 70. Food intake and body composition measurements continued weekly. The average weekly food intake increased by 67.6% in AL offspring and by 31.8% in eTRF offspring after switching to HFHS, both of which exceed energy needs for adult mice [41]. Similar to the findings on chow, with HFHS, there were no major differences between eTRF and AL offspring in body weight (Figure 3(a), *p*_diet_ = 0.99), fat mass (Figure 3(b), *p*_diet_ = 0.65), or lean mass (Figure 3(c), *p*_diet_ = 0.47). Therefore, offspring of eTRF and AL experienced similar changes in body composition in response to overnutrition. Cumulative HFHS consumption was comparable between females and males (*p*_sex_ = 0.72) and maternal restriction groups (Figure 3(d), *p*_diet_ = 0.72). Feeding efficiency, a ratio comparing food intake to stored fat and lean mass, was greater in males than in females, which is consistent with the NCD period (Supplemental Figure 2B, *p*_sex_ = 0.00023). However, unlike the NCD period, efficiency was indistinguishable between eTRF and AL offspring (*p*_diet_ = 0.93).

We repeated an ITT and GTT after 10 weeks of HFHS feeding. During the ITT, there was a significant interaction between sex and diet using mixed linear-effect modeling (Figure 3(e), p_sex:diet_ = 0.03). Female eTRF had a similar response to insulin, with less than a 1 mg/dL difference from their AL counterparts (*p*_diet_ = 0.85), but male eTRF offspring tended to be more insulin sensitive with 25 mg/dL lower glucose than AL males (*p*_diet_ = 0.17). It could also be true that females were more resilient to changes from HFHS. These findings were confirmed by calculating AUC where eTRF females showed no difference in AUC compared to AL females (Figure 3(f), *p*_diet_ = 0.20), while eTRF males had 20.4% lower AUC than AL males (*p*_diet_ < 0.0001). The initial rate of glucose decline (not pictured) was greater in females than in males (*p*_sex_ = 0.029), but there were no differences between eTRF and AL offspring (*p*_diet_ = 0.23). The trend toward insulin sensitivity from the ITT was not explained by fasting blood glucose, as females had 23% lower fasting blood glucose than males (*p*_sex_<0.0001), but was similar between eTRF and AL offspring within the same sex (Figure 3(i), *p*_diet_ = 0.83). Glucose tolerance tests in Figure 3 also show a significant effect of interaction (*p*_sex:diet_ = 0.011), although now in the opposite direction. During GTT, eTRF males trended toward glucose intolerance with an average of 53 mg/dL higher glucose than AL males during the course of the experiment (*p*_diet_ = 0.14). This was not observed in female eTRF offspring, which had similar blood glucose during the GTT compared to AL females (*p*_diet_ = 0.61). The GTT AUC showed interaction between effects of sex and treatment (Figure 3(h), (*p*_sex:diet_ < 0.0001)). AUC was 5% lower in eTRF females (*p*_diet_ = 0.07) but was 13.5% higher in eTRF male offspring than in AL (*p*_diet_ < 0.0001). Taken together, these tests suggest eTRF results in males who experience glucose intolerance and insulin sensitivity, whereas females are more resilient to glycemic changes after gestational eTRF. Given that we cannot explain glucose intolerance in males via reduced insulin sensitivity, we next evaluated insulin secretion.

After noticing eTRF males developed glucose intolerance after HFHS diet exposure in both cohorts, we sought to explore cohort 2 more closely for insulin secretion defects, via an *in vivo* glucose-stimulated insulin secretion (GSIS) assay (Figure 3(j)). Females had lower levels of insulin than males (*p*_sex_ < 0.0001). There was a nonsignificant trend toward lower insulin levels in eTRF compared to AL offspring of both sexes (*p*_diet_ = 0.071). Females had similar increases in insulin in response to glucose injection, 139% in AL versus 137% eTRF. Male AL offspring had a 48% increase in insulin, whereas this was just an 18% increase for eTRF males. There was no interaction between sex and maternal restriction (*p*_sex:diet_ = 0.064). Females have 94% greater fold-change insulin secretion in response to glucose challenge than male offspring (*p*_sex_ = 0.0027), but there was no impact of maternal restriction on fold change secretion (*p* = 0.85, Figure 3(k)). Male and female offspring of eTRF dams had lower baseline insulin values than those of AL dams, which we believe resulted in the similarity of fold-change insulin secretion between maternal restriction groups. This study was not conclusive as it had a lower sample size and failed to reach statistical significance but could indicate that insulin secretion is modestly impaired in male eTRF offspring after HFHS challenge in males.

## 4. Discussion

This study is the second to describe the long-term effects of gestational eTRF on offspring health and the first to describe their response to a high-fat, high-sucrose diet challenge. We find a minimal effect of eTRF during gestation while male and female offspring are consuming a chow diet through early adulthood. However, after prolonged HFHS diet feeding and advanced age, glucose intolerance develops in adult male progeny. Taken together, results from insulin and glucose tolerance testing and exploratory GSIS after HFHS feeding suggest modest reduction in insulin secretion between eTRF and AL males. Although the latter was exploratory and did not reach statistical significance, the other study of gestational (12-hour) TRF of a chow diet in rats also found evidence of glucose intolerance and insulin sensitivity in the offspring of TRF dams [19]. However, two studies were not completely consistent. Most notably, they found impaired GSIS in both male and female without exposure to HFHS. The modest reduction of insulin at baseline during GSIS in eTRF offspring may contribute to the modest insulin sensitivity seen after HFHS feeding in the current study, and this is consistent with others noting modest improvements in insulin sensitivity in females [19]. There were reductions in insulin secretion in response to high glucose in male and female dark cycle-fed islets after gestational TRF, suggesting this may be a contributing mechanism for metabolic disruption in our model of gestational TRF.

Other studies that focus on lighting manipulations during gestation highlight similar effects among adult offspring. Perinatal exposure to chronodisruption in rats and mice also resulted in mild phenotypes of glycemic dysmetabolism [15, 16, 42, 43]. This is similar to the current study, as this effect is present without reductions in birth weight or litter size [16, 42]. Taken together, these data imply that the chronological timing of multiple zeitgebers can impact perinatal health outcomes.

Comparing the current study with other studies utilizing HFHS diets and TRF demonstrates some consistencies in glycemic outcomes. Fasting insulin can be lowered [33–36, 44], similar to our findings, and resulting HOMA-IR can be improved with TRF [35, 44, 45]. Our finding that fasting blood glucose is unchanged in eTRF mice is consistent with other groups examining TRF with HFHS [33, 44, 45]. Some differences in current studies are not reflected in the literature, such as elevated food intake while on NCD in female offspring exposed to eTRF *in utero*, and were not seen in the other longitudinal analysis of offspring health following gestational TRF [19]. Studies of adult mice pairing TRF and HFHS report reduced food intake in TRF groups [37, 46] or equivalent caloric intake when matched by diet [34–36, 47]. This could indicate a compensatory response in the female offspring resulting from eTRF *in utero*. Interestingly, this did not result in differing body weight or composition, suggesting that this increased food intake is matched by decreased caloric extraction or increased energy expenditure in these mice.

The phenotype in male offspring from time-restricted feeding bears resemblance to animal models of adverse intrauterine development, where glucose intolerance in resultant offspring can be a common phenotype. First, as described by Barker et al., offspring who were deprived of nutrition *in utero* were more likely to develop chronic, nutrition-related disease in adulthood [48]. Undernutrition [49–51], overnutrition [52, 53], placental insufficiency [54, 55], and chronodisruption [15, 16, 42, 56] during pregnancy have all been reported to induce offspring glucose intolerance. The extent to which male-predominate phenotypes and female resilience to changes are difficult to deduce as many groups either study male offspring exclusively [51, 57] or analyze males and females together [50, 58].

Although we did not evaluate insulin conclusively in the current study, glucose intolerance in adverse intrauterine development models has been found to co-occur with insulin-related abnormalities in the offspring, including lower insulin content in the pancreas [50], lower basal circulating insulin levels [58], impaired insulin secretion [51, 59], and reduced beta cell mass [60]. However, in the present study, we find modest improvement in male insulin sensitivity in adulthood in male offspring exposed to gestational eTRF. This finding is similar to the previous study where females exposed to gestational TRF had greater rates of glucose disappearance during insulin tolerance testing [19]. We believe that insulin sensitivity during high-fat, high-sucrose diet feeding in eTRF males could be attributed to having low basal levels of insulin compared to AL males in our model. This could result in peripheral tissues being more sensitive to insulin action despite apparent insulin secretion impairment at the level of the pancreas. However, without formal experimental evaluation of islet form and function or peripheral insulin signaling, we cannot conclude this is the mechanism for these phenotypic differences in eTRF offspring.

In contrast to the previous study and some models of adverse intrauterine environment in pregnancy, we did not observe major metabolic differences between restricted and unrestricted offspring until an HFHS diet challenge occurred in adulthood. Our findings bears similarity to the phenotype of adult offspring born to dams that experienced chronodisruption during pregnancy metabolic consequences only manifested after 12 months of age [16, 42]. This could suggest that gestational eTRF may be relatively safe to practice in the context of a healthful diet or absent a second challenge. However, it also suggests that in the context of unhealthy diet patterns, adult offspring may be ill-equipped to adapt to high-calorie food environments, leading to metabolic dysfunction. These studies differ both in the age of onset and duration of food restriction that are required to initiate glucose intolerance in offspring of TRF dams which also may explain these differences. Modeling of this dietary strategy remains incomplete, so translation to human clinical populations is not possible at this time. The similarity of the present study to those using diverse gestational stressors suggests that restriction of the total time spent eating in pregnant dams is a novel dietary component that can have a lasting impact on the metabolic health of offspring. This recommends further research on this novel component in the diet as a modulator of maternal and child metabolic health outcomes.

Although we have not investigated offspring pancreatic tissues, we hypothesize that alterations in the development of the pancreas may underlie the susceptibility of males for glucose intolerance and modest insulin sensitivity in eTRF offspring after HFHS feeding. This is confirmed by one study of early postnatal exposure to TRF, which found that adolescent males who were fed TRF the first 4 weeks after weaning developed smaller islets of Langerhans and higher blood glucose than those fed AL [47]. Therefore, future studies of gestational or developmental eTRF should examine islet size, pancreatic beta cell mass, and insulin secretion to investigate the mechanism of offspring glucose intolerance more conclusively.

This study and the conclusions to be made from it have some limitations. First, the model of gestational eTRF may have resulted in differences in maternal behaviors that were not noted by the study team and therefore could play a part in the effects seen in the offspring. Second, we assessed the effect of a dietary insult in young adulthood by switching all animals to HFHS. As such, disentangling the effect of an HFHS diet from that of aging and gestational eTRF is not possible in this model. It is also worth noting that several metabolic diseases are highly linked to age, and while our study ended at approximately six months of age, mice can live much longer under laboratory conditions, typically 26–30 months. As metabolic, physical, cognitive, and other phenotypes that do not appear until toward the end of the mouse's lifespan were not detectable, and we look forward to future studies on geriatric mice treated *in utero* with eTRF. Finally, although we see a robust effect on glucose intolerance, we were not powered to conclusively establish lower insulin secretion in male eTRF offspring in adulthood and have not yet evaluated islet size or beta cell mass to determine the mechanisms driving the worsening of glucose tolerance in adulthood in male mice or the resilience of female mice. Furthermore, while dams were manipulated simultaneously, we cannot rule out that our eTRF treatment induced other confounding differences that we have not accounted for, including potential maternal stress or chronodisruption. Our model used healthy, nonobese dams; therefore, we cannot extend the effects of the current study to the context of metabolic syndrome, diabetes, or obesity during pregnancy. Future work should prioritize assessing the pancreas and islets in larger samples and with higher resolution so that more in-depth conclusions can be drawn.

There are many strengths in this study. Among them is the use of a preclinical model which facilitates consistency when compared to existing literature and allows for careful control of diet, genetics, and environment throughout gestation, which would be impossible at this point in a human trial. Further strengths include the long follow-up period for gestational exposure, controlling for the effect of litter size, repeated measurement of body composition, and food intake measurements over the life course in the resultant offspring. Finally, the inclusion of both male and female offspring in the study, as many metabolic assessments of TRF either focus exclusively on the effects of the regimen in males [35, 36] or female mice [33, 34], is a strength.

## 5. Conclusion

Offspring who are exposed to eTRF of NCD *in utero* have similar body composition, glucose tolerance, and insulin tolerance in early adulthood in both males and females. Gestational eTRF resulted in male impairments in glucose tolerance in adulthood only after chronic HFHS feeding, whereas females appeared resilient to and did not develop differences. This occurs without a increase in body weight, fat mass, or food intake compared to age-matched AL males. More research is warranted to understand the mechanisms that underlie this novel phenotype.

## Data Availability

All raw data and reproducible code are available for this manuscript at https://github.com/BridgesLab/Developmental-Obesity.

## Supplementary Materials

Supplementary Materials Body weight gain and food intake during gestation for dams of the described offspring did not differ between assigned dietary groups (Supplemental Figure 1). The efficiency of male and female offspring in converting consumed food into body tissues while on a normal chow diet was lower in eTRF animals (NCD, Supplemental Figure 1A) but was no different between groups while on a high-fat, high-sucrose diet (HFHS, Supplemental Figure 1B). Supplementary figure 2 estimates the feeding efficiency (the extent to which food intake is converted to mass) in each group, showing that female offspring have lower feeding efficiency than males, and that overall there is a significant reduction of gestational eTRF intervention on offspring feeding efficiency in the NCD but not HFD phase.
