# Supplementary figures and images for: Gestational Early-Time Restricted Feeding Results in Sex-Specific Glucose Intolerance in Adult Male Mice
Source: J Obes. 2023 Sep 29;2023:6666613. doi: 10.1155/2023/6666613 (PMC10558268; doi:10.1155/2023/6666613)

**Supplemental Figure 1:**

**A**

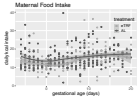

**B**

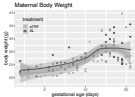

Supplement: Supplementary Materials — Body weight gain and food intake during gestation for dams of the described offspring did not differ between assigned dietary groups (Supplemental Figure 1). The efficiency of male and female offspring in converting consumed food into body tissues while on a normal chow diet was lower in eTRF animals (NCD, Supplemental Figure 1A) but was no different between groups while on a high-fat, high-sucrose diet (HFHS, Supplemental Figure 1B). Supplementary figure 2 estimates the feeding efficiency (the extent to which food intake is converted to mass) in each group, showing that female offspring have lower feeding efficiency than males, and that overall there is a significant reduction of gestational eTRF intervention on offspring feeding efficiency in the NCD but not HFD phase. [file 6666613.f1.zip › Mulcahy-eTRFandoffspring-Supplemental_Figure_1.pdf]

## Supplemental Figure 2:

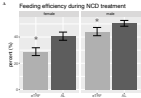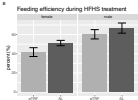

Supplement: Supplementary Materials — Body weight gain and food intake during gestation for dams of the described offspring did not differ between assigned dietary groups (Supplemental Figure 1). The efficiency of male and female offspring in converting consumed food into body tissues while on a normal chow diet was lower in eTRF animals (NCD, Supplemental Figure 1A) but was no different between groups while on a high-fat, high-sucrose diet (HFHS, Supplemental Figure 1B). Supplementary figure 2 estimates the feeding efficiency (the extent to which food intake is converted to mass) in each group, showing that female offspring have lower feeding efficiency than males, and that overall there is a significant reduction of gestational eTRF intervention on offspring feeding efficiency in the NCD but not HFD phase. [file 6666613.f1.zip › Mulcahy-eTRFandoffspring-Supplemental_Figure_2.pdf]
